# Preparation and characterization of a curcumin nanoemulsion gel for the effective treatment of mycoses

**DOI:** 10.1038/s41598-023-49328-2

**Published:** 2023-12-20

**Authors:** Adel Al Fatease, Ali Alqahtani, Barkat A. Khan, Jamal Moideen Muthu Mohamed, Syeda Ayesha Farhana

**Affiliations:** 1 Department of Pharmaceutics, College of Pharmacy, King Khalid University, 62529 Guraiger, Abha, Saudi Arabia; 2 Department of Pharmacology, College of Pharmacy, King Khalid University, 62529 Guraiger, Abha, Saudi Arabia; 3 Drug Delivery and Cosmetics Lab (DDCL), GCPS, Faculty of Pharmacy, Gomal University, D.I. Khan, 29050 Pakistan; 4 Faculty of Pharmacy and BioMedical Sciences, MAHSA University, Bandar Saujana Putra, 42610 Jenjarom, Selangor Malaysia; 5 Department of Pharmaceutics, Unaizah College of Pharmacy, Qassim University, 51911 Unaizah, Saudi Arabia

**Keywords:** Health care, Medical research

## Abstract

Fungal infections of skin including mycoses are one of the most common infections in skin or skins. Mycosis is caused by dermatophytes, non-dermatophyte moulds and yeasts. Various studies show different drugs to treat mycoses, yet there is need to treat it with applied drugs delivery. This study was designed to prepare a bio curcumin (CMN) nanoemulsion (CMN-NEs) for transdermal administration to treat mycoses. The self-nanoemulsification approach was used to prepare a nanoemulsion (NE), utilizing an oil phase consisting of Cremophor EL 100 (Cre EL), glyceryl monooleate (GMO), and polyethylene glycol 5000 (PEG 5000). Particle size (PS), polydispersity index (PDI), zeta potential (ZP), Fourier transform infrared (FTIR) spectrophotometric analysis, and morphological analyses were performed to evaluate the nanoemulsion (NE). The in vitro permeation of CMN was investigated using a modified vertical diffusion cell with an activated dialysis membrane bag. Among all the formulations, a stable, spontaneously produced nanoemulsion was determined with 250 mg of CMN loaded with 10 g of the oil phase. The average droplet size, ZP, and PDI of CMN-NEs were 90.0 ± 2.1 nm, − 7.4 ± 0.4, and 0.171 ± 0.03 mV, respectively. The release kinetics of CMN differed from zero order with a Higuchi release profile as a result of nanoemulsification, which also significantly increased the flux of CMN permeating from the hydrophilic matrix gel. Overall, the prepared nanoemulsion system not only increased the permeability of CMN but also protected it against chemical deterioration. Both CMN-ME (24.0 ± 0.31 mm) and CMN-NE gel (29.6 ± 0.25 mm) had zones of inhibition against Candida albicans that were significantly larger than those of marketed Itrostred gel (21.5 ± 0.34 mm). The prepared CMN-NE improved the bioavailability, better skin penetration, and the CMN-NE gel enhanced the release of CMN from the gel matrix on mycotic patients.

## Introduction

Numerous human diseases are caused by fungal infections, commonly referred to as mycoses. Mycoses can vary in severity, ranging from superficial infections affecting the skin's stratum corneum to extensive infections involving the spleen, liver, brain, lungs, and kidneys. Individuals with acquired immunodeficiency syndrome, those undergoing cancer therapy, recipients of organ transplants, and patients undergoing major surgery constitute a growing population susceptible to invasive fungal infections. The likelihood of invasive fungal infections is high within each of these patient groups. The repertoire of opportunistic fungal pathogens infecting these patients continues to expand, in parallel with the increasing population at risk. Identifying severely invasive mycoses at an early stage and achieving successful treatment can be challenging. Ongoing extensive research is focused on the development of novel strategies for the detection and treatment of invasive fungal diseases^1^. All across the world, superficial mycoses are frequent. It is estimated that between 20% and 25% of people on the planet are impacted, and the frequency is rising.1. The dermatophytes are the main culprits, and the specific species involved differ according on the location^2^.

Curcumin (CMN), a natural polyphenolic compound typically derived from *Curcuma longa Linn*, exhibits potent anti-inflammatory properties when administered orally or topically. CMN hinders the metabolism of cyclooxygenase, arachidonic acid, lipoxygenase, nuclear factor-kB, and the activation of pro-inflammatory cytokines, even at high doses with low inherent toxicity. Nevertheless, the utility of CMN is constrained by its poor solubility in aqueous solutions at neutral or acidic pH levels, significant degradation in alkaline environments, and swift breakdown into inactive metabolites during first-pass metabolism, resulting in restricted bioavailability in the systemic circulation^3^.

Several approaches have been explored to boost the biological efficacy of CMN, such as complex interactions or formation with macromolecules, chemical derivatization, and the utilization of nano drug delivery systems. In recent years, numerous studies have explored the incorporation of nanotechnology in CMN, aiming to enhance its effectiveness and formulation. The encapsulation of CMN within nanoscale carrier systems, such as nanoemulsion, notably enhances its bioavailability.

Nanoemulsions (NE) serve as exceptionally stable carriers safeguarding active substances from harsh conditions^4^. Prior investigations indicate that an oil-in-water (o/w) type NE, incorporating curcumin (CMN) with mean droplet sizes spanning from 80 to 620 nm, can augment the anti-inflammatory properties of CMN while preserving its stability^5^. Nevertheless, these studies did not specifically address transdermal delivery. Transdermal delivery through patches or gels is an interesting alternative to topical administration for achieving local or systemic effects^6^. This method has several advantages for patients, including improved compliance and reduced side effects due to the absence of first-pass metabolism, allowing for steady blood levels for extended periods. Unfortunately, CMN has poor efficacy due to its limited skin penetration^7^. Recently, a stable CMN gel was prepared using 15% alcohol to dissolve CMN completely in the gel phase, which may help increase its efficacy^8^.

In addition, dimethylsulfoxide (DMSO) was needed to improve the release of CMN from the gels^9^. Compared to traditional chemical skin penetration, a solvent-free topical carrier based on drug entrapment in oil-in-water (o/w) emulsion-type droplets of submicron particles is more effective in terms of percutaneous absorption. This method has no adverse effects compared to enhancers such as organic solvents, which are typically associated with skin irritation, toxicity, and sensitization^10^. Additionally, the unique large hydrophobic core of o/w and high solubility for aqueous-insoluble topically active substances have been made possible by submicron-sized emulsion droplets, which also enhance the delivery of water, an excellent moisturizer, to the skin^11^. This study presents a more efficient and effective transdermal delivery method for CMN. To improve bioavailability by increasing skin penetration and CMN stability, this work also aims to enhance the release of CMN from the gel matrix.

## Materials and methods

### Materials

The supplier of curcumin (CMN) was obtained from SRL Pvt. Ltd, Maharashtra, India. Cremophor EL 100 (Cre-EL), also known as Polyoxyl 35 Castor Oil (BASF Corporation, Mumbai, India), glyceryl monooleate (GMO), and polyethylene glycol 5000 (PEG 5000), and Propylene glycol (PG) procured from S.D. Fine Chem. Pvt. Ltd, Mumbai, India. Triethanolamine, Glycerine, methylparaben, and propylparaben were purchased from Hi Media, Mumbai, India. Analytical grade chemicals, reagents, and internally prepared double distilled water were used in this research.

### Preparation of micoemulsion (CMN-ME) and nanoemulsion (CMN-NE)

Six formulations of CMN-ME were prepared by stirring CMN (10, 25, 50, 100, 250, and 500 mg), GMO (32% w/v), and Cre-EL (8% w/v) for 30 min, followed by the dropwise addition of water to prepare an emulsion that served as a standard. The CMN-NE was produced by mixing oil, CMN, surfactant, and co-surfactant on a magnetic stirrer (REMI2) for 1.5 h at 600 rpm^12^. Cre-EL, PEG 5000, and GMO were used in a ratio of 8:1:1. CMN was applied in different amounts (10, 25, 50, 100, 250, and 500 mg) towards 10 g of the oil phase (triethanolamine). To complete the emulsification process, additional sonication for 30 min was performed using a sonicator (Sonics & Materials VCX 750, Inc., Newtown, USA) with a pulse rate of 6/2 s and an amplitude of 35%. Nanoemulsion was prepared at a 5:1 ratio, and double-distilled water was added to the oil phase while gently stirring^13^.

### Dynamic light scattering analysis (DLS)

The hydrodynamic droplet size (DS), zeta potential (ZP), and polydispersible index (PDI) of CMN-ME and CMN-NE were measured by DLS utilizing Zetasizer (Nano ZS90; Malvern Instruments Ltd., Malvern, UK), according to the previous method described by Qushawy et al. (2022)^14^. NE has previously diluted appropriately with Milli-Q water to provide better-focusing positions and for lighting.

### Incorporation efficiency % % IE %

A direct method was employed to measure the % IE of CMN-ME and CMN-NE. To extract CMN, 5 mL of dimethyl sulfoxide (DMSO) was added to 10 mL of the supernatant obtained after centrifuging the nanoemulsion at 14,000 rpm for 20 min. The amount of CMN in the DMSO phase was measured using a UV–visible spectrophotometer (Agilent Cary 60 UV–Vis Spectrophotometer, USA). The following equation was used to calculate the percentage of CMN in the CMN-NE^8^.

$$\mathrm{\% IE}=\frac{\mathrm{CMN encapsulated in a nano oil globule}}{\mathrm{CMN added to NE}}\times 100$$

### Fourier transform infrared (FTIR) spectrophotometric analysis

To evaluate potential interactions (structural variations) among the CMN, Cre-EL, GMO, PEG 5000, CMN-ME, blank NE (B-NE), and CMN-NE, FTIR analysis was conducted^15^. In an FTIR spectrophotometer (JASCO/FTIR-6300, Japan), IR spectra of the powdered materials were recorded using the KBr disc method spanning the wavenumber range of 4000–400 cm^−1^ with a scan velocity of 1 cm.s^−1^^16^.

### Morphology of CMN-ME and CMN-NE by SEM

The CMN-ME and CMN-NE preparation morphology was determined by scanning electron microscope (SEM) (JSEM-6360LA, JEOL, Tokyo, Japan). The sample was dropped on aluminum stubs, dried, and then sputter coated with platinum by the auto-fine platinum coater before imaging^17^. Samples were scanned under vacuum conditions at 15 kV acceleration voltage at room temperature.

### Preparation of CMN-ME and CMN-NE gels

The gel base was prepared by dispersing 5% w/v of Viscolam AT100P in distilled water while stirring using a Remi RQ-124 A/D Direct Drive Stirrer (Maharashtra, India)^8^. The CMN-ME and -NE gels were prepared by the dispersion of 0.1% w/v of the CMN with continuous stirring at 500 rpm. Propylene glycol (15% w/v) and glycerine (5% w/v) were added as humectants. Propylparaben (0.05% w/v) and methylparaben (0.2% w/v) were added as preservatives. The pH was then adjusted to 6–7 using triethanolamine^18^.

### Evaluation of CMN gel

The prepared gels were assessed visually, and the pH, viscosity, drug content, and in-vitro permeation study.

#### Determination of pH

To determine the pH of the freshly prepared gels (CMN-ME and CMN-NE), 1 g of each formulation was mixed with 20 mL of distilled water and subjected to a digital pH meter to determine the pH value. The determinations were done in triplicates, and the mean ± SD was computed^19^.

#### Determination of gel viscosity

The viscosity of the prepared gels (CMN-ME and CMN-NE) was measured using spindle number CC 14 on a Brookfield viscometer R/S + RHEOMETER (Brookfield Inc., MA, USA). In brief, 100 mg of the gel sample was placed on the sample holder, and the spindle was lowered for 5 min to equilibrate. At room temperature, the spindle revolved at a shear rate of 10/s and a speed of 10 rpm, and the corresponding viscosity was measured. The measurements were performed in triplicate, and the mean ± SD was determined^20^.

### Determination of drug content

The drug content was determined by transferring 100 mg of each gel (CMN-ME and CMN-NE) into a clean volumetric flask (100 mL) and filling the remaining space with distilled water. The contents were agitated for two h before being filtered and spectrophotometrically measured at 425 nm. The measurements were taken three times to ensure accuracy, and then the mean and standard deviation were calculated^21^.

#### In vitro permeation study

As mentioned in a prior report, the penetration of CMN was investigated using a modified vertical diffusion cell and activated dialysis membrane^22^ (M.W cutoff of 12,000; dialysis membrane 110 (LA 395); Hi-media, Mumbai, India). Before the process, the dialysis membrane was moistened and put among the receptor and donor compartments. The system included a donor compartment containing 1 g of gel containing CMN-ME or CMN-NE and a receptor compartment containing 66 mL of phosphate buffer (PBS) pH 7.4 at 37 °C temperature and slowly stirring at a speed of 100 rpm. There were 0.951 cm^2^ of possible diffusion area between the compartments. Throughout 24 h, accurately 1 mL of the diffusion medium was taken via the diffusion cell sampling port at specified intervals of 5, 10, 15, 30, and 45 min and 1, 1.5, 2, 3, 4, 5, 6, 7, 8 and 24 h). One mL of freshly prepared buffer was replaced in the receptor compartment. As previously mentioned, UV-Spectrophotometer was used to determine the amount of CMN permeated. The experiments were carried out in triplicate, and mean ± SD was calculated. The permeated amount of CMN was plotted against time. The slope of the linear part of the figure was used to compute the transdermal drug flow. The permeation kinetics was established as zero and first order, Korsmeyer-Peppas and Higuchi and as described by the previous method^23^.

#### Anti-fungal activity

*Candida Albicans* was used to study the antifungal activity of the control, and a specific sample of CMN-ME anwas d CMN-NE loaded into the gels. Mixing 9.9 mL of liquid broth (excluding agar) with 0.1 mL of the fungal culture suspension, they were then cultured in an incubator (Remi instruments cooling incubator, Mumbai, India) at 25 °C for 24 h^24^. One mL of inoculated liquid broth comprising a suspension of a fungal culture was placed into the sterile petri plates holding the solidified agar growth medium. The plate was then rotated in both the clockwise and counterclockwise directions to equally disseminate the inoculum throughout the firm agar surface. Using a sterile cork-borer to make wells in the middle of the plates, each well (6 mm inner diameter) was carefully filled with either 0.1 mL of plain gel, CMN-ME gel, and CMN-NE gel. In order to facilitate fungal development, the plates were then incubated in the incubator for three days at 25 °C. By measuring the zones surrounding the formulations where microbial growth was inhibited, antifungal efficacy was determined. They underwent a thorough antifungal investigation in an aseptic setting and were measured on a mm scale.

#### Stability study of gel

Both room temperature (25 ± 1 °C) and 40 ± 1 °C with 75% RH was used to examine the stability of CMN-ME and CMN-NE gels during storage for 28 days. Every day, samples were taken, and checked for drug content.

#### Data analysis

The data were stated as mean value standard deviation for each experiment carried out in triplicate. The one-way ANOVA and Student's t-test were used for statistical data analysis. It was decided that a value of *p* < 0.05 was statistically significant.

## Results and discussion

Self-nano emulsification is only possible when a specific combination of oils, surfactants, and co-surfactants is used. In o/w NEs, choosing the proper oily phase is crucial since it affects the choice of additional constituents. The oily phase for the preparation of NE was typically the oil with the highest solubility for the desired medication. This facilitates the most excellent possible drug loading^25^. Naturally, a surfactant alone did not reduce the oil–water interfacial tension enough to produce a nanoemulsion; therefore, an amphiphilic short-chain molecule must be added. For instance, a surfactant or cosurfactant can be used to reduce the surface tension to almost nil. By adding more variability to the interfacial film and breaking the liquid crystalline phases that form when the surfactant film is excessively rigid, co-surfactants penetrate the surfactant monolayer^26^.

**Figure 1:**
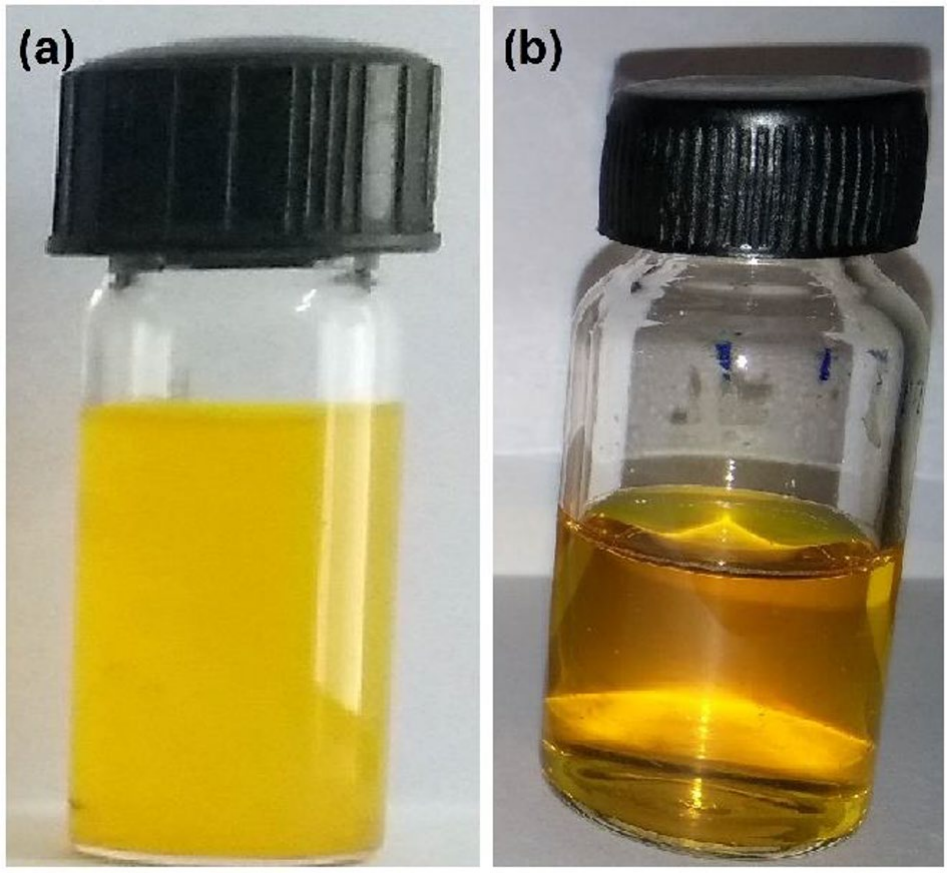
The appearance of CMN-ME and CMN-NE containing an equal quantity of CMN.

The self-emulsification method prepared standard CMN-ME and CMN-NE with different CMN concentrations. The prepared formulations were examined for droplet size, PDI, ZP, and IE%. Figure 1 compares the visualization of CMN-ME (Fig. 1a) with CMN-NE (Fig. 1b) at the same CMN concentration. The NEs differ from microscale emulsions in several intriguing physical ways. MEs often have a white appearance due to visible solid light scattering. In contrast, as the structures of NEs are far smaller than the visible light wavelengths, they typically have an optically transparent appearance.

### The droplet size, PDI, and ZP

**Figure 2:**
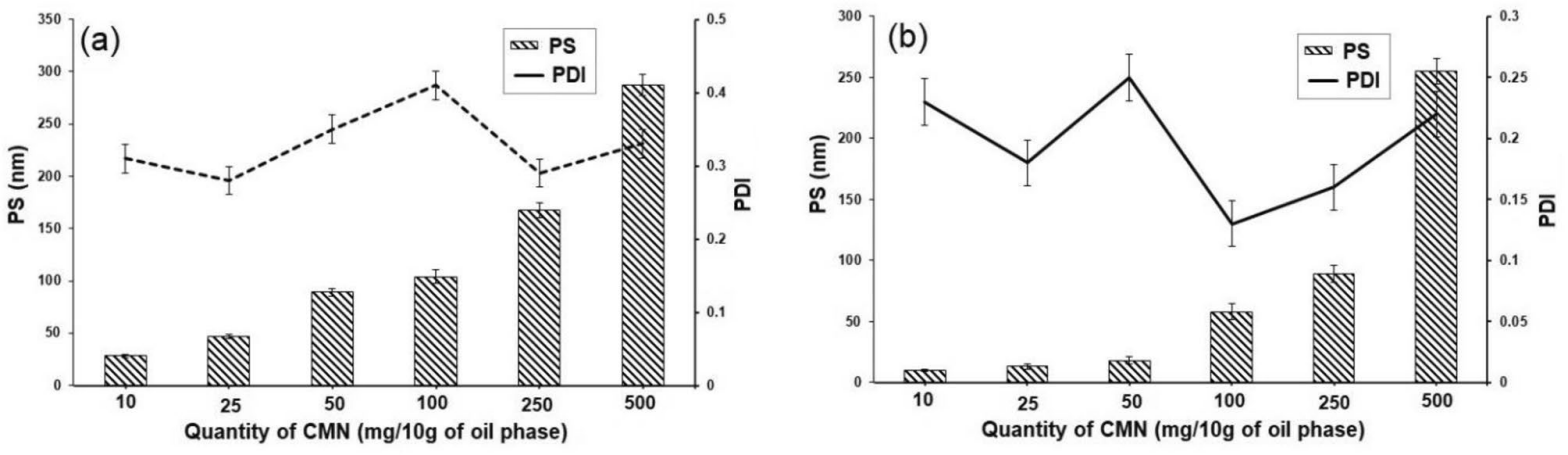
The characterization of different formulations of (**a**): CMN-ME and (**b**): CMN-NE.

As shown in Fig. 2, it was found that the droplet size was increased by increasing the amount of CMN up to 500 mg. The suitability of CMN-NE was fixed based on spontaneity, which was determined as the average droplet size, ZP, and PDI of CMN-NE were 90.0 ± 2.1 nm, − 7.4 ± 0.4, and 0.171 ± 0.03 mV, respectively. The quantity of CMN has increased with the size of NE up to 500 mg in the CMN-ME. Conversely, the globule size (214 ± 7.23 nm), ZP (− 6.8 ± 0.7), and PDI (0.289 ± 0.08 mV) exhibited an overall increase in all the preparations of CMN-MEs compared to CMN-NEs. These outcomes could be attributed to CMN, which potentially possesses amphiphilic properties. This implies that a portion of the drug molecules can integrate themselves as a spacer into the surfactant monolayer at the oil–water interface, leading to an enlargement in droplet size^27^. Another reason for the increase in average droplet size of NE as drug content increased could be the increased amount of drug in the lipophilic core of surfactants. Moreover, in cases where an excess of the drug was utilized and remained undissolved, the observed enlargement in size may be associated with the creation of drug aggregates on the surface of oil droplets^28^. Similar findings were reported by Sakeena et al. (2011), who, in their preparation of ketoprofen NEs, observed an increase in droplet size with escalating drug concentration^29^. Additionally, Anuchapreeda et al. (2012) noted that the droplet size of curcumin nanoemulsion grew with an increase in the curcumin quantity, ranging from 15 to 240 mg^30^.

The PDI values of all prepared CMN-ME and CMN-NE were less than 0.5, indicating the homogeneity of droplet size^31^. According to ZP, it was found that the prepared CMN-ME and CMN-NE possessed a negative charge, which may be attributed to the breaking of the fatty acid ester in Cre-EL into a negatively charged free fatty acid^32^. The low value of ZP may be due to the high concentration of the non-ionic surfactant. Non-ionic surfactants are known to contribute to lower ZP values compared to other types of surfactants. It would be beneficial to explore this further through the adjustments to the formulation to determine the optimal balance between the non-ionic surfactant concentration and ZP values. This insight will not only help us understand the current observations better but also guide us in refining the formulation for desired properties^33^. Also, the functional group’s interaction in CMN with the functional groups of other components in the formula may cause the various ZP values at different concentrations of CMN in nanoemulsion^34^.

The PDI reflects the heterogeneity or uniformity of droplet sizes within a sample. High PDI values suggest a broader size distribution, often indicative of particle aggregation or a non-uniform dispersion. The occurrence of aggregation can be influenced by various factors, such as the formulation conditions, concentration of particles or molecules, and the presence of stabilizing agents. To address this issue and achieve a more uniform size distribution, further optimization of the formulation or adjustments to experimental conditions may be necessary.

### The % IE

**Figure 3:**
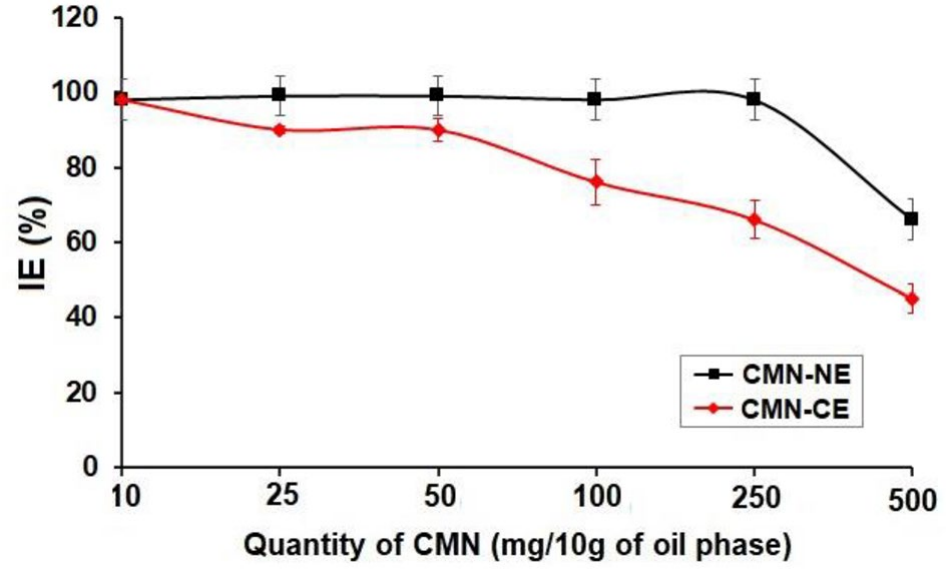
The % IE of CMN concentration on the effectiveness of CMN-NE and CMN-ME (mean ± SD, n = 3).

According to the results represented in Fig. 3, it was found that the IE% was decreased by increasing the amount of CMN in both CMN-ME and CMN-NE. Which may be attributed to the limited capacity of oil droplets. Anuchapreeda et al. (2012) found that the IE% of curcumin nanoemulsion was decreased by increasing the curcumin amount from 15 to 240 mg^30^.

### FTIR outcome

**Figure 4:**
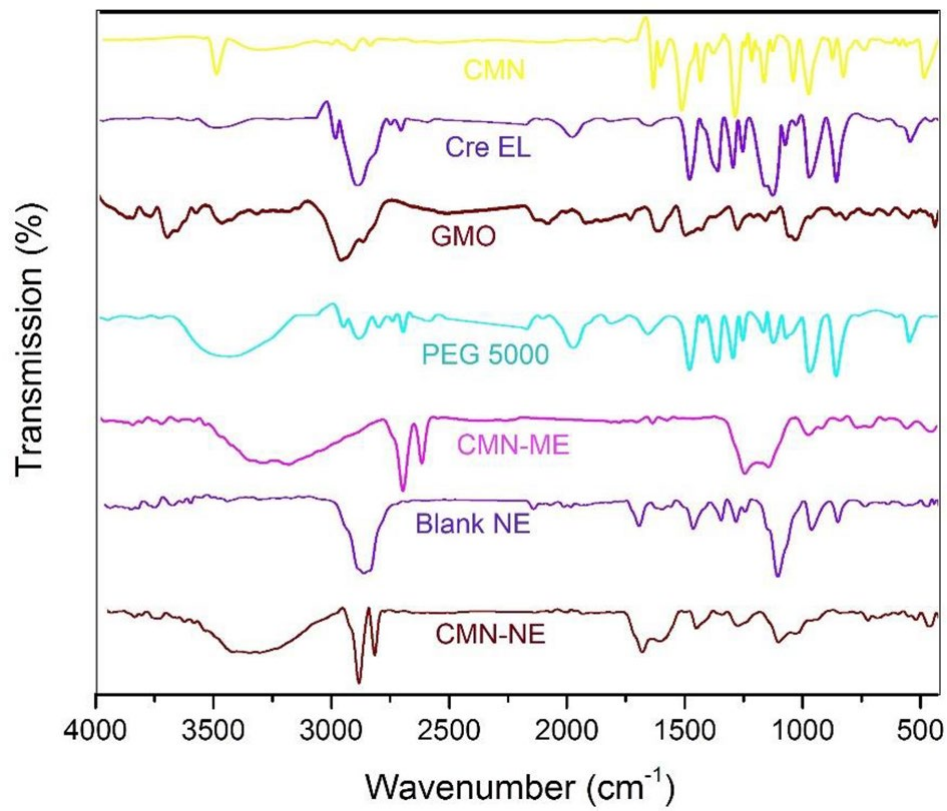
FT-IR spectra of pure CMN, Cre-EL, GMO, PEG 5000, CMN in GMO, physical mixture, blank NE, and CMN-NE.

As shown in Fig. 4, The spectrum of curcumin displayed a sharp absorption peak at 3510 cm^−1^ due to the phenolic –OH stretching vibration. The strong peak at 1742.37 cm^−1^ corresponding to the C=O group and 1597.56 cm^−1^ was attributed to the symmetric aromatic stretching vibration. The sharp peak at 1507.1 cm^−1^ was due to C=C vibrations. The sharp peak at 1,455 cm^−1^ was due to phenolic C–O, while the enolic C–O peak appeared at 1278 cm^−1^. The peak at 1,025 cm^−1^ was attributed to the asymmetric stretching of C–O–C. The peak at 721 cm^−1^ was the C–H vibration of the aromatic ring^35,36^.

The IR spectrum of Cre-EL displayed a broad band at 3436 cm^−1^ corresponding to the –OH group, a peak at 2926 cm^−1^ due to C–H stretch, and small absorption band between 1715–1730 cm^−1^ characteristic of C=O stretch for esters. A rise at 1642 cm^−1^ due to stretching band of C=C, the band from 1101 cm^−1^ was attributed to the C–O stretch from alcohols, and the broad absorption of 636 cm^−1^ to = C–H bend^37^. The IR spectrum of GMO showed a characteristic peak at 2861 cm^−1^, 2882 cm^−1^ due to C–H stretching, and peak at 1463 cm^−1^ due to C=C, respectively^38^. The IR spectrum of PEG 5000 displayed sharp peaks at 2889, 1630, and 1111 cm^−1^, corresponding to the stretching vibrations of the C-H, C=O, and C–O. Also, peaks at 1465 and 1340 cm^−1^ region represent the C-H deformation vibrations and peaks at 1284 and 1242 cm^−1^ due to the O–H bending vibrations^39^. The IR spectra of MN-ME, blank NE, and CMN-NE revealed the absence of interaction between CMN and the other ingredients.

### The surface morphology of CMN-ME and CMN-NE

**Figure 5:**
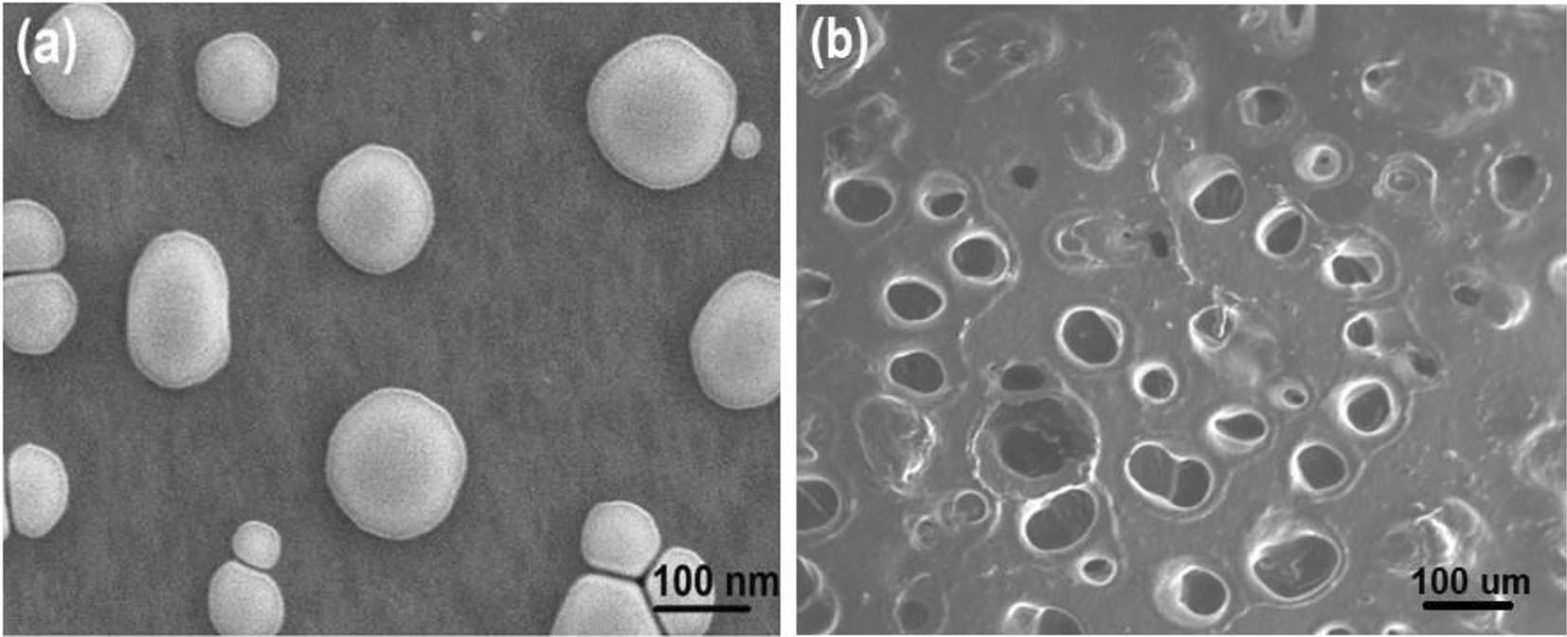
SEM image of prepared (**a**) CMN-NE and (**b**) CMN-ME.

The surface morphology and shape of the prepared CMN-ME and CMN-NE were examined using SEM. As shown in Fig. 5a, the droplets of CMN-NE appeared spherical, uniform, and monodispersed. The spherical shape and absence of aggregation indicate the physical stability of the nanoemulsion. This stability may be attributed to the presence of surfactant and cosurfactant. In contrast, CMN-ME, as shown in Fig. 5b, appeared irregular in shape with non-identical droplet sizes. These results are in good agreement with de Oliveira Filho et al.^40^ who examined the surface morphology of carnauba wax micro and nanoemulsions using SEM.

### Characterization of CMN gels

The prepared CMN gels (CMN-ME and CMN-NE gels) appeared homogeneous dispersion. As represented in Table 1, the pH values of the prepared CMN-ME and CMN-NE gels were 6.2 and 6.9, respectively. These values were acceptable for application on the skin. The viscosity of CMN-ME and CMN-NE gels was 2800 ± 85.40 and 2200 ± 120.12 Cps, respectively, indicating good extrudability and spreadability. The drug content in the prepared gels was 98.5 ± 5.77 and 98.7 ± 6.62 for CMN-ME and CMN-NE gels, respectively.

**Table 1:** CMN-NE and CMN-ME permeation flux of gel (mean SD, n = 3).

Formulation	pH	Viscosity (Cps)	Drug content (%)	Flux (mg/(cm^2^ h))
CMN-ME gel	6.2 ± 0.31	2800 ± 85.40	98.5 ± 5.77	0.754 ± 0.008
CMN-NE gel	6.9 ± 0.25	2200 ± 120.12	98.7 ± 6.62	1.764 ± 0.061

### In vitro permeation of CMN from CMN-ME and CMN-NE gels

**Figure 6:**
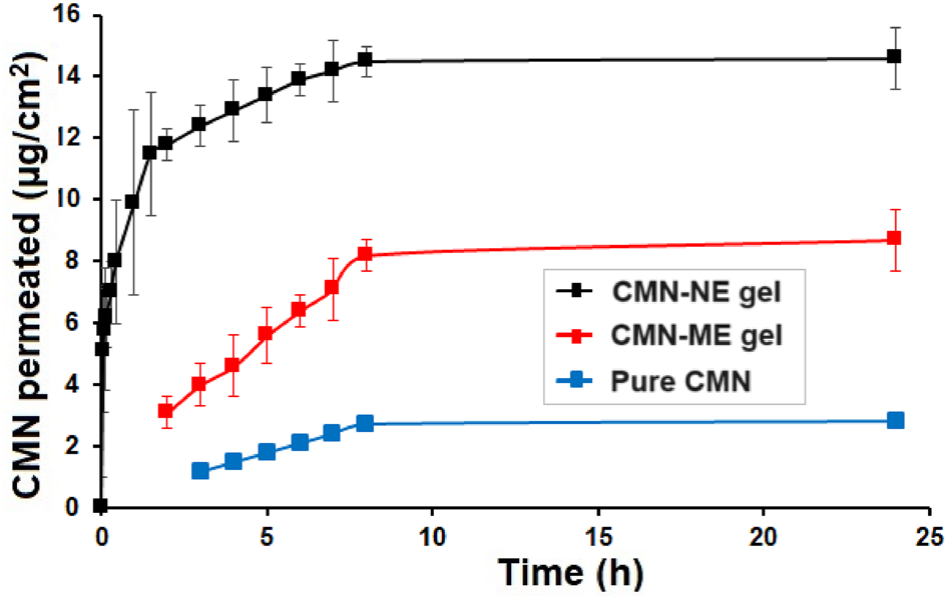
Cumulative in vitro CMN permeation of CMN-NE and CMN-ME gel (mean + SD, n = 3).

CMN-ME and CMN-NE gels underwent in vitro permeation study utilizing a modified vertical diffusion cell using a dialysis membrane with PBS; pH 7.4 as dissolution medium. Figure 6 illustrates the entire CMN release from pure CMN, CMN-ME and CMN-NE gel, it was found that the cumulative amount of CMN permeated were 3.12 ± 0.24, 8.76 ± 0.51 and 14.66 ± 0.72 µg/cm^2^ for 24 h, respectively. It was found that the amount permeated from CMN-NE gel was higher than CMN-ME gel and pure CMN which may be due to the smaller size of the oil droplets of NE.

The principal ingredient in gels moisturizes the skin, allowing the stratum corneum cells to expand, resulting in the widening of drug channels and increased cumulative permeation. The cumulative amounts of CMN penetrated versus the time curve were used to calculate the permeation flux. As shown in Table 2, the statistical assessment of the flux in a 24 h testing period reveals that CMN-NE gels produce a flux (*p* < 0.05) that is higher than that of pure CMN and CMN-ME gel. This is consistent with earlier research, which demonstrated a notable enhancement in the cumulative CMN permeated by the NE gel^41^.

**Table 2:** CMN-NE and CMN-ME gel in vitro permeation kinetics.

Formulation	Model	r^2^	Equation
CMN-ME gel	Zero-order	0.997	y = 0.853x + 1.5120
	First order	0.979	y = 0.076x + 0.414
	Korsmeyer-Peppas	0.979	y = 0.7112x + 0.313
	Higuchi	0.986	y = 4.002x + 2.201
CMN-NE gel	Zero-order	0.961	y = 1.152x + 4.989
	First order	0.902	y = 0.061x + 0.802
	Korsmeyer-Peppas	0.974	y = 0.299x + 1.009
	Higuchi	0.992	y = 4.108x + 2.302

Interactions between CMN-NE and the stratum corneum, change both the polar and nonpolar routes, leading to higher penetration rates. By increasing the solubilization of the lipids found in the stratum corneum and increasing membrane fluidity, changes to the tight junction characteristics of the stratum corneum also promote penetration^42^. Direct drug penetration from the CMN-NE droplets to the stratum corneum is another potential method. It hypothesized that nanosized droplets in CMN-NE in the continuous phase can convey the drug through the epidermal barrier and can migrate easily into the stratum corneum, resulting in an improved transfer of the drug from NE and this is in line with other researchers also demonstrated^8^. Earlier it is found that CMN in polymer matrix enhances its applicability as anti-cancer agent as well as in photodynamic therapy^43^.

A burst in the first release of CMN from the gel is also depicted in Fig. 4. The polymer that was utilized, the gels polymeric matrix, prepares slack channels within the network, which results in a rapid initial release of the CMN. The penetration kinetics were established using the total amount of CMN that passed through the dialysis membrane^44^. For transdermal transport kinetics, four kinetic equations are suitable: zero order, first order, Korsmeyer-Peppas, and Higuchi. Plots of the cumulative CMN zero order (permeated vs time), first order (log cumulative CMN permeated vs time), Korsmeyer-Peppas (log cumulative CMN permeated vs log time), and Higuchi (log cumulative CMN permeated vs square root of time) were made, respectively. Each of these permeation kinetics correlation coefficients was determined and compared (Table 2). The value of the correlation coefficient revealed the permeation kinetic that fit the data the best. The permeation characteristics of CMN-ME and CMN-NEs followed, respectively, zero-order and CMN released often follows Higuchi model, as observed earlier^45^, as shown in Table 2. The concentration of the active compound and polymer both impacted the variation in penetration kinetics of the active compound. However, the observed variance in kinetics in this work can only be attributed to the type of CMN, i.e., in crystalline or dissolved states, as the composition of a gel including CMN-ME and CMN-NE was analogous.

### The efficacy of mycoses

Given that *Candida albicans* (MTCC No. 227) is the most prevalent dermatophyte that causes mycoses, several studies recommended using it to assess in vitro antifungal efficacy^3,37^. The agar diffusion technique was employed to investigate the antifungal efficacy of plain gel, CMN-ME gel, and CMN-NE gel, in comparison with the commercial product (Itrostred gel). The clear circles observed around the dishes represent the ZOI, which expands with formulation efficiency. Interestingly, the unmedicated gel (control) against *Candida albicans* demonstrated a mean ZOI of 6.2 ± 0.16 mm.

As represented in Table 3, the mean ZOI for CMN-ME gel was 24.0 ± 0.31 mm, while the mean ZOI for CMN-NE gel was 29.6 ± 0.25 mm, which was substantially greater than the mean ZOI for the commercial preparation Itrsostred gel (21.5 ± 0.34 mm). This might be due to the larger release and diffusion potential of formulation CMN-NE gel^46^. Note that vesicles have been successfully used in the topical treatment of mycoses employing liposomal, transfersomal, and ethosomal terbinafine. The findings show that skin penetration enhancers with novel CMN-NE are a potential fungal delivery mechanism that can be used in clinical trials on mycoses patients.

**Table 3:** Antifungal activity of various formulations.

Preparation	ZOI* (mm)
Plain gel	6.2 0 ± 0.16
CMN-ME gel	24.0 ± 0.31
CMN-NE gel	29.6 ± 0.25
Marketed Itrostred gel	21.5 ± 0.34

*Each value represents mean, n = 3 ± SD.

### Stability study of CMN gel

A 28-day stability study of CMN-ME and CMN-NE gels was conducted at 25 and 40 °C with 75% RH. The examination focused on the CMN content in the CMN-ME and CMN-NE gel. As shown in Fig. 6, the decrease in CMN during the storage period was higher in the CMN-ME gel than in the CMN-NE gel. This difference may be attributed to the improved solubility of CMN in aqueous gels, while also providing protection to CMN from deterioration.

**Figure 7:**
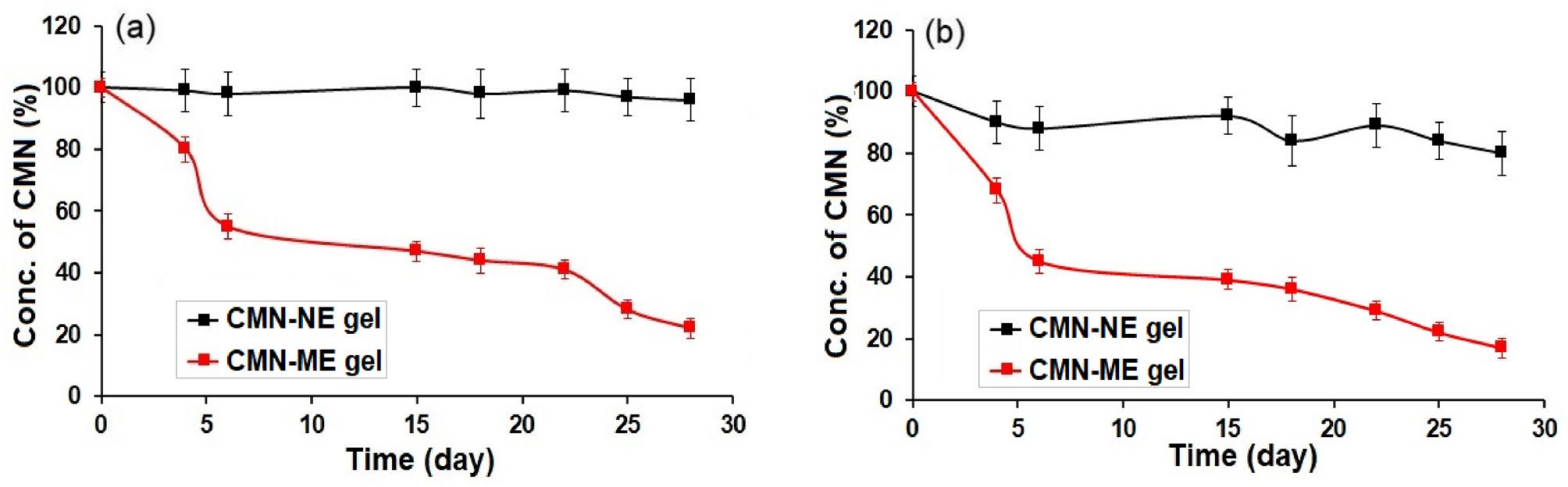
(**a**) Degradation profiles of CMN from CMN-NE and CMN-ME gel stored at 28 days (**a**) 25 ± 1 °C and (**b**) 40 ± 1 °C with 75% RH (mean + SD, n = 3).

After being stored for 28 days at either room temperature or 40 °C, the pH and viscosity of all the gels examined in this study remained largely unchanged. The gel viscosities ranged from 2200 to 2800 cps for CMN-ME and CMN-NE gels, ranging, respectively. Indicate that they are both comparatively easy to pour and sufficiently viscous to adhere to the skin and maintain physical stability over time. All of the gels tested had a pH between 6.2 and 6.9 for CMN-ME and CMN-NE gels, respectively, which was acceptable. Although the human skin surface has a pH between 5.5 and 5.9, using gels with a pH as low as neutral did not irritate the skin. Gels with CMN-NE exhibited greater stability in terms of CMN content in the gel under both conditions compared to gels with CMN-ME. In Fig. 7, the stability of nanoemulsion gels was three times better at room temperature and 4.5 times better in a humidity chamber than that of CMN-ME gels. These improvements were statistically significant (*p* < 0.05), indicating that nanoemulsions containing surfactant and co-surfactant play a major role in enhancing CMN stability in the gels.

## Conclusions

Due to its low skin permeability and various issues with oral administration, the topical application of CMN poses challenges. An ideal transdermal delivery strategy for mycoses treatment with CMN is the CMN-NE gel. CMN was successfully loaded, resulting in improved physicochemical stability, a longer shelf life, and enhanced skin permeability when combined with Cre-EL: PEG 5000: GMO (8:1:1). Encapsulating CMN in a nanoemulsion holds promise for transdermal delivery, as it requires this permeability to achieve the necessary therapeutic dose. The developed CMN-NE gel meets the best requirements for topical application, spreading readily and exhibiting maximum slide and drag. The CMN-NE gel demonstrated superior antifungal efficacy compared to the commercial gel, and the nanosized formulations increased drug permeability while prolonging retention at the site of action.

## Data Availability

The datasets used and/or analysed during the current study are available from the corresponding author on reasonable request.
